# Traditional vs. AI-generated meteorological risks for emergency predictions

**DOI:** 10.3389/frai.2025.1545851

**Published:** 2025-03-24

**Authors:** Naoufal Sirri, Christophe Guyeux

**Affiliations:** FEMTO-ST Institute, UMR 6174 CNRS, University of Franche-Comté, Belfort, France

**Keywords:** firefighters intervention, feature selection, prediction, XGBoost, Large Language Model (LLM)

## Abstract

This study aims to analyze and examine in-depth the feature selection process using Large Language Models (LLMs) to optimize firefighter prediction performance. Although features from reliable sources are known to significantly aid predictions, their accuracy may be limited in critical situations requiring rigorous prioritization. Therefore, the focus was placed on meteorological risks for a comparative diagnosis between their extraction from Météo France and those generated by LLMs across various dimensions. Given the crucial role of meteorological risks as key informational sources for decision-making, this study explores the impact of feature extraction methods related to these risks on predicting firefighter interventions over nine years, from 2015 to 2024. Annual reports on firefighter activities in France highlight the growing influence of weather-related risks, underscoring the urgent need for precise and actionable meteorological information to support rapid and effective emergency response strategies. The methodology implemented involved comprehensive data preparation, an in-depth analysis of feature extraction through different approaches, and their evaluation from multiple perspectives. This required leveraging machine learning models such as XGBoost, Random Forest, and Support Vector Machines (SVM) to assess and analyze prediction results based on two feature spaces: F1 (including general features and meteorological risks extracted from Météo France) and F2 (including general features and meteorological risks generated by LLMs). The results revealed that models trained with the F2 feature space consistently demonstrated superior performance. Notably, annual improvements were observed, particularly for high and very high intervention activities. However, the use of the F2 space proved less effective for low intervention activities and underperformed compared to F1 during the summer season. In conclusion, this work presents a concrete methodology for forecasting and enhancing resource management, accelerating firefighter response times, and ultimately contributing to life preservation by reducing the risk of failure during critical incidents.

## 1 Introduction

The use of artificial intelligence to anticipate the needs of emergency services has become a crucial tool for managing urgent situations and accurately forecasting operational requirements. This is particularly important in sectors like environmental protection and public safety. Enhancing the predictive performance of firefighter interventions and optimizing resource allocation relies on extracting features for machine learning models from reliable and precise data sources. This is particularly relevant for meteorological risk features, where inaccurate estimations can lead to serious consequences, such as insufficient resource deployment and delayed response times. Assessing features extracted from reliable sources over a nine-year period (2015-2024) to develop robust predictions presents significant challenges. Using diverse approaches to identify meteorological risk features, whether derived from traditional sources such as Météo France or generated by Large Language Models (LLMs) (Raiaan et al., 2024), this study aims to evaluate the outcomes from multiple perspectives. The objective is to select the most effective feature set for predictive modeling. The objective is to identify the best feature set for predictive modeling. These approaches facilitate a thorough comparative analysis and inform decision-making regarding the most suitable feature extraction method based on seasonal variations or firefighter activity levels.

Previous research has made significant strides in feature extraction and selection for prediction tasks across various domains, including fire intervention management. Recent studies have taken this further by employing generative AI to identify relevant features. For instance, Raweh et al. (2018) proposed a hybrid approach combining feature selection and extraction to enhance cancer prediction using DNA methylation data, effectively addressing challenges related to high dimensionality and noise. Similarly, Malekipirbazari et al. (2021) demonstrated that randomly reducing the number of instances before feature reduction could maintain high classification performance while minimizing data requirements and computational time. On the other hand, Kabongo et al. (2024) highlighted the critical role of context selection in improving the accuracy of Large Language Models (LLMs) for data extraction, outperforming traditional methods and reducing errors. Additionally, Lin et al. (2024) introduced an effective method for selecting representative samples for few-shot fine-tuning of LLMs, achieving enhanced performance with only 2% of the data and reducing computational costs by 97%.

However, limited research has focused on predicting firefighter interventions, and no study to date has specifically explored the application of generative AI, particularly Large Language Models (LLMs), in this field. For instance, Cerna et al. (2020) employed machine learning techniques to enhance resilience and efficiency through optimized firefighter deployment strategies in France. Similarly, Cerna et al. (2022) proposed leveraging natural language processing techniques to extract features from weather bulletins, achieving high accuracy (80-92%) in predicting intervention peaks caused by rare events. Finally, in relation to the topic addressed in this paper, Sirri and Guyeux (2024c) assessed the reliability of predictions concerning firefighter interventions, aiming to optimize resource utilization and response times. They also conducted several separate studies analyzing the impact of specific factors such as air quality, solar activity, river height, and weather conditions on fire intervention activities, as detailed in Sirri and Guyeux (2024a), Sirri and Guyeux (2024e), Sirri and Guyeux (2024d), and Sirri and Guyeux (2024b), respectively.

Our research team is fully committed to this initiative by adopting an innovative approach: analyzing and evaluating meteorological risk features using various methodologies, including extraction from traditional sources such as Météo-France and data generated by Large Language Models (LLMs). This analysis is enriched by comprehensive historical datasets, offering a wealth of information on various aspects of firefighter interventions. The primary aim of this study is to assess the impact of feature extraction methods on improving the accuracy of firefighter intervention predictions. To achieve this, a hypothesis and its corresponding prediction are presented as part of this research effort:

- Hypothesis: Integrating meteorological risks generated by LLMs into predictive modeling provides a superior alternative to traditional sources (e.g., Météo-France) for optimizing firefighter intervention predictions.
- Prediction: Models incorporating features generated by LLMs will outperform those utilizing meteorological risks from traditional sources regarding predictive accuracy, especially for high-intervention activities.

To ensure the precision of this study, a well-structured methodological framework was established, meticulously adhering to its nuanced details as outlined below. Section 2 provides a comprehensive overview of the methodologies and materials utilized across the various experiments. The study's progression is detailed in Section 3, which carefully presents the results obtained through this research. Section 4 delves into a thorough discussion, analyzing the findings in depth to address the initial research question. This section offers an extensive evaluation and diagnosis of the outcomes, emphasizing relevant implications and interpretations. Finally, Section 5 concludes the study by summarizing key discoveries, highlighting significant contributions, and proposing avenues for future research. This methodological framework facilitated a systematic exploration of generative AI's capabilities in extracting meteorological risk features. Through a comprehensive comparison of two approaches from multiple perspectives, this analysis remained well-organized, rigorous, and critically focused throughout the investigation.

## 2 Materials and methods

### 2.1 Data handling

#### 2.1.1 Sources and data extraction

In this study, the data collection phase played a crucial role. A comprehensive dataset was used, provided by the Fire and Rescue Services of Doubs, France. This dataset encompasses 334,536 intervention records spanning from January 1, 2015, to October 31, 2024. Each entry includes details such as start and end timestamps, location, duration, and type of intervention. To enrich this dataset with potentially relevant features, variables were collected from diverse sources.

**Figure 1:**
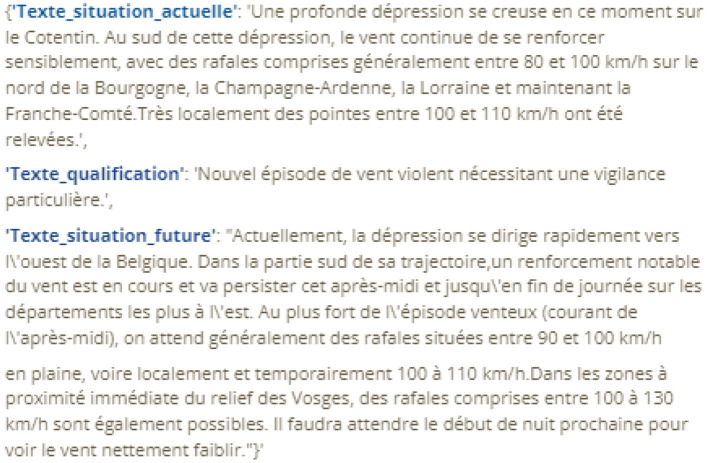
Example of a weather alert bulletin (in French).

- General Features: Basic features included school periods, holidays, solar activity, air quality data, and epidemiological information. Notably, epidemiological data were collected on diseases such as influenza, chickenpox, and acute diarrhea, sourced from the Sentinel Network (Sentinel, 2024). Astronomical features, including lunar phases, moonrise, and distance between the Earth and the Moon, were added to evaluate their impact on night visibility (Astral, 2024; Skyfield, 2024). Satellite data from NASA's VIIRS and MODIS missions were also integrated, leveraging different wavelengths and resolutions to assess wildfire propagation in specific regions (Nasa Viirs, 2024; Nasa Modis, 2024). Calendar-related features captured details such as holidays, academic breaks (Ministry of National Education, 2024), and time-related attributes like hours, days, weeks, months, and years. Additionally, solar activity metrics, such as sunspot area, the 10 cm radio flux, and the sunspot count, were included (Nasa Solar Activity, 2024). Air quality variables, including fine particles, ozone, and nitrogen oxides, were obtained from monitoring stations in the region (ATMO-BFC, 2024).
- Meteorological Features: In a second phase, meteorological vigilance bulletins from Météo France covering the Doubs region were included. These reports detailed various weather risks with assigned color codes and included textual bulletins to enhance the overall meteorological understanding (refer to Table 1, Figure 1) (Vigilance-France, 2024).

**Table 1:** Categorization of meteorological risk by alert levels.

**Weather risk**	**Green**	**Yellow**	**Orange**	**Red**
Wind	76,544	1,204	2,602	0
Precipitations	75,769	1,560	3,021	0
Thunderstorms	72,863	3,853	2,745	889
Snow-ice	79,885	0	465	0
Heatwave	79,061	324	965	0
Extreme cold	80,248	0	102	0

Green indicates no risk, Yellow indicates low risk, Orange indicates high risk, and Red indicates very high risk.

The complete dataset was systematically enriched, integrating firefighter intervention data with these diverse features from multiple sources (refer to Table 2). This comprehensive dictionary serves as a robust foundation for the analysis conducted in this study.

**Table 2:** Distribution of variables across feature families.

**Family of features**	**Number of variables**
Calendar	9
Temporal	6
Holidays and academic breaks	8
Epidemiological	4
Astronomical	8
Solar activity	12
Air quality	11
Earth images	2
Meteorological	6

#### 2.1.2 Data refinement and processing

**Figure 2:**
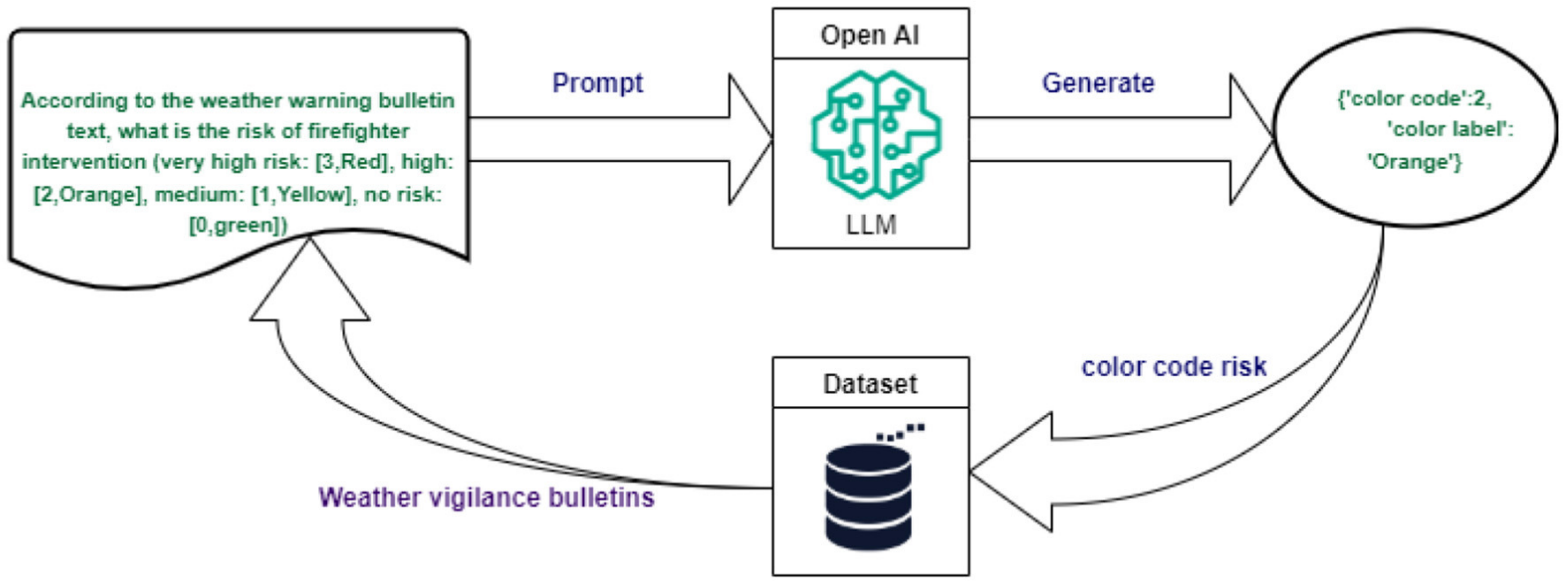
Integration of AI-Driven features for enriching meteorological risk data.

To optimize the input data for the machine learning model, several pre-processing techniques were applied. First, the “StandardScaler” technique from the Scikit-learn library Pedregosa et al. (2011) was employed to normalize numerical variables. This method adjusts the data distribution to achieve a mean of zero and a unit variance, ensuring consistent scaling across variables. The normalized features included the year, time of day, and statistics for diseases such as chickenpox, influenza, acute diarrhea, and the Earth-Moon distance. In addition, the TargetEncoder technique (Target-Encoder, 2024) was applied to encode categorical variables. This approach replaces each category with the mean of the corresponding target variable. Features such as the day of the week, year, month, and public holidays were encoded using this method. Additionally, meteorological risk features were enriched by employing OpenAI's large language model (LLM). The LLM was solicited through a specific prompt designed to analyze weather vigilance bulletins and extract structured color-coded risk information. Specifically, the implementation leveraged the LangChain framework to send prompts such as: “According to the weather warning bulletin text, what is the risk of firefighter intervention (very high risk: [3, Red], high: [2, Orange], medium: [1, Yellow], no risk: [0, Green])?” Based on textual descriptions of weather warnings (see example in Figure 1), the LLM generated responses in JSON format, for example: “color code”: 2, “color label”: “Orange”. These outputs were used to populate the meteorological risk feature in the dataset. The integration of these AI-generated features significantly enhanced the dataset by providing additional, highly relevant variables (refer to Figure 2).

To facilitate effective classification, the original target variable representing the number of emergency interventions was transformed into an ordinal qualitative scale ranging from 0 to 3, based on percentile thresholds:

- 0: Low intervention activity
- 1: Moderate intervention activity
- 2: High intervention activity
- 3: Very high intervention activity

The percentile thresholds were determined to ensure a balanced distribution of cases across the categories, allowing for better model discrimination and interpretability. Specifically, these thresholds were derived from the dataset's distribution of intervention counts, ensuring that each category reflects a meaningful and distinct level of activity. The relevance of these thresholds is rooted in their alignment with operational needs, where different risk levels correspond to actionable thresholds for resource allocation in emergency response scenarios.

#### 2.1.3 Exploratory data insights

A thorough analysis of the dataset, extracted from multiple sources, is crucial to uncover relevant observations that can provide insights for this research. On average, 32,000 fire intervention events are recorded annually, equating to more than four interventions per hour. It was observed that the number of interventions has been consistently increasing each year due to various factors such as demographic aging, significant population growth, the effects of climate change, and the closure of small hospitals.

**Figure 3:**
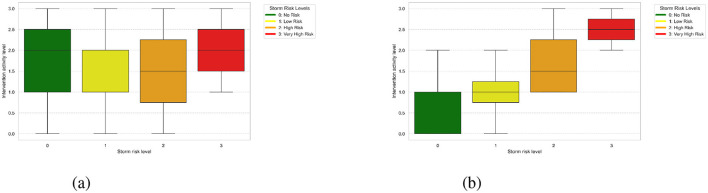
Intervention activity distribution across storm risk levels. **(A)** Storm risk levels from Météo Fr. **(B)** Storm risk levels from LLM.

**Figure 4:**
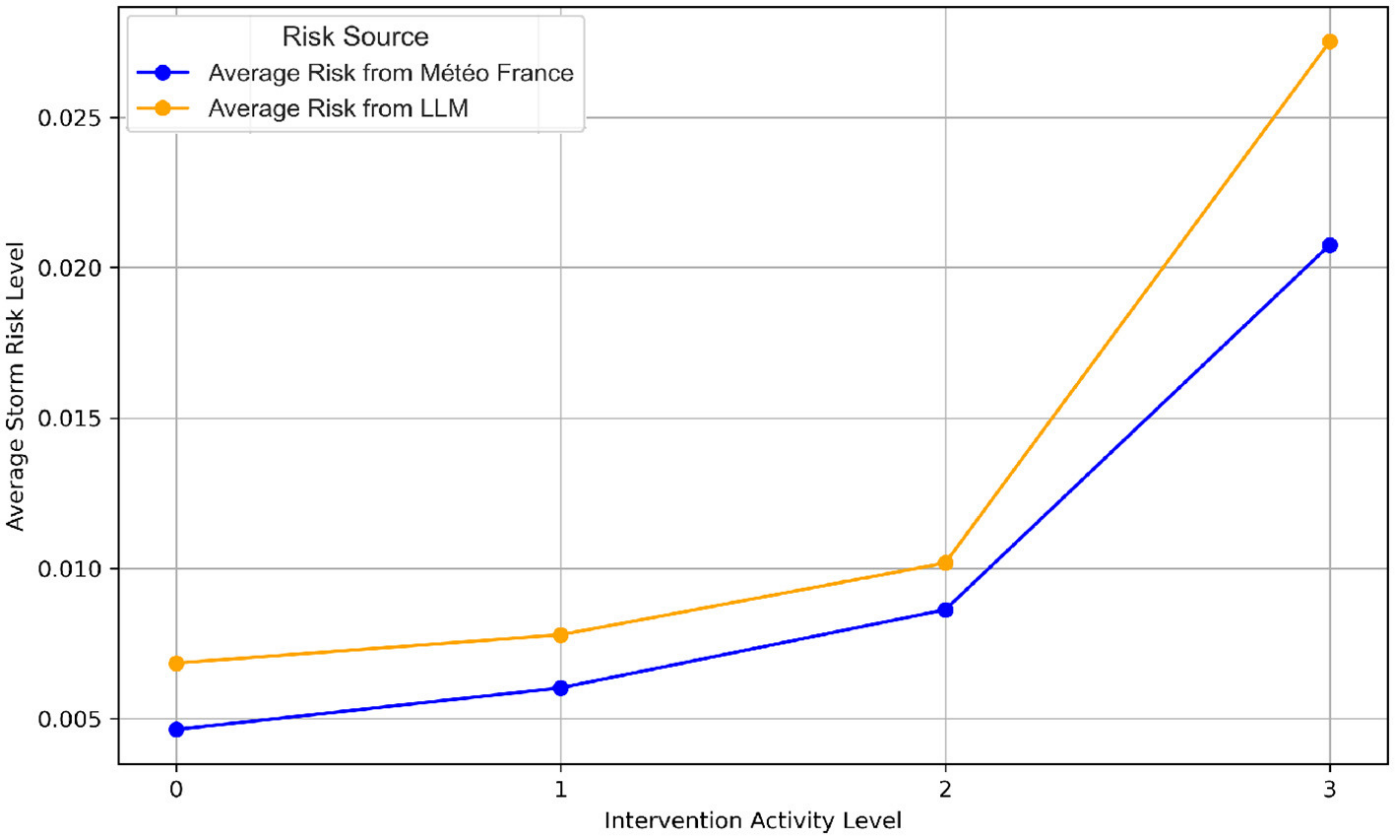
Average storm risk levels: Météo France vs. LLM by intervention activity.

In this study, the analysis of meteorological risks played a vital role in identifying and optimizing key features for predicting firefighter interventions. For instance, Figures 3A, B illustrate the distribution of storm risks from Météo France compared to those generated by the Large Language Model (LLM). It is evident that the risk levels from Météo France are largely aligned with those of the LLM, which shows more dispersed values. This discrepancy is likely due to the fact that Météo France uses calibrated thresholds based on meteorological expertise, while the LLM-derived risks are based on text analysis, leading to a wider variability due to linguistic nuances and the absence of direct calibration. Also, it is clear that the dominance of low-risk for both Météo-France and LLM sources confirms that such scenarios are the most prevalent in the dataset. Additionally, a correlation analysis was performed to evaluate the relationship between meteorological risk features and firefighter activity levels. The results revealed no significant correlation between the meteorological risk levels (from either Météo-France or the LLM) and the intervention activity levels. This lack of correlation suggests that while meteorological risks are valuable for understanding context, they may not directly influence the intensity of firefighting activities without the inclusion of additional situational variables. Furthermore, Figure 4 visualizes the average storm risks from Météo-France and those derived from the LLM in relation to firefighter activity. While both sources exhibit a similar trend, Météo-France's risks are slightly lower on average, reflecting its standardized and more conservative approach to risk evaluation. In contrast, the LLM's broader range of values indicates its sensitivity to the diversity of textual expressions in the input data.

Feature importance analysis using XGBoost further substantiated these findings. Features such as storms, wind, snow, and extreme cold derived from the LLM source were shown to have higher importance compared to their Météo-France counterparts, potentially due to the nuanced information encoded in textual risk descriptions. Conversely, heatwaves and precipitation from Météo-France were identified as more impactful, likely reflecting the strength of standardized meteorological assessments in these specific contexts.

In conclusion, while exploratory data analysis revealed that low-risk levels dominate the dataset and that meteorological risks from both sources lack direct correlation with firefighter activity, their inclusion remains valuable. They provide essential contextual features that complement other predictors, thereby enhancing the overall performance of the predictive models.

### 2.2 Feature engineering and predictive models

#### 2.2.1 Feature engineering

Traditionally, all features in a dataset are included during model training, assuming each feature significantly contributes to building an optimal model. However, including all features has significant drawbacks. Some may exhibit strong correlations with others, while some may have limited usefulness. This can lead to suboptimal generalization or the inclusion of redundant information. Additionally, using all features can increase computational time without necessarily improving model performance (Garreta and Moncecchi, 2013). Therefore, selecting a limited or optimized set of features can improve model performance. In this study, the “feature importance” technique was employed, which assigns a score to each variable based on its relevance to the target variable (Zien et al., 2009). However, this technique was applied only to the general features. Meteorological risk features were analyzed separately in two feature spaces:

- F1: General features + Météo-France risk features.
- F2: General features + LLM-generated risk features.

This study used the following feature selection techniques:

1. Pearson and Kendall Correlation Coefficients: features with an absolute correlation of 0.4 or less with the target variable were excluded.
2. Chi-Square selector: the Chi-Square test was used to evaluate the dependency between each feature and the target variable, and then select the top “K” best variables.
3. Light Gradient Boosting model [LightGBM (Ke et al., 2017)]: this model was employed with specified hyperparameters (learning rate = 0.1, objective = regression, metric = RMSE, number of leaves = 2^9^, maximum depth = 9, and number of estimators = 120,000). Early stopping was implemented after 10 iterations.

**Figure 5:**
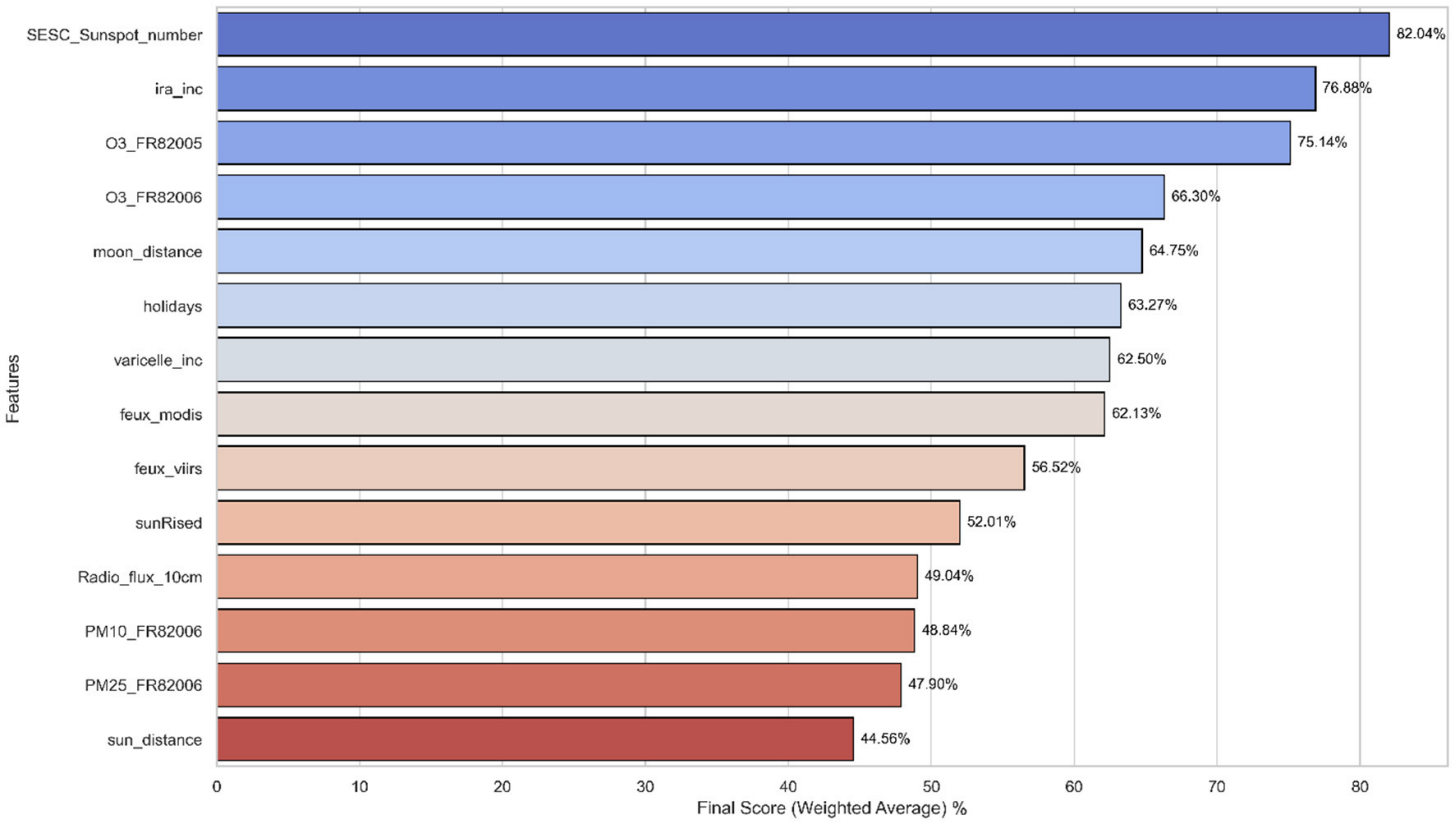
Top 14 feature importance based on combined methodologies.

The final feature selection process produced a coherent set of features, emphasizing those identified as important across most methodologies and those with high scores. Figure 5 presents the top 14 features with the highest combined importance scores derived from the three methodologies.

#### 2.2.2 Predictive models

To achieve meaningful results, three models were selected: Random Forest (RF) (Breiman, 2001), Extreme Gradient Boosting (XGBoost) model (Chen and Guestrin, 2016), and Support Vector Machine (SVM) (Cortes, 1995). These models were chosen for their robustness, ability to handle complex datasets, and widespread use in classification problems. The combination of these models leverages their complementary strengths: XGBoost for capturing complex relationships, Random Forest for its resistance to noise, and SVM for its precision on well-separated data.

1. Random Forest (RF): Random Forest is an ensemble learning method that combines multiple decision trees, each trained on random samples of the dataset. The final prediction is the average (for regression) or the majority vote (for classification) of all trees, reducing the risk of overfitting. Random Forest is particularly effective for handling categorical variables and provides reliable estimates of feature importance (Equation 1).
  (1) $$\begin{array}{l} {ŷ}_{i}=\frac{1}{K}\overset{K}{\underset{k=1}{\sum }}f_{k}\left({\text{x}}_{i}\right) \end{array}$$
  ŷ_*i*_ represents the predicted value for observation *i*.
  *K* is the total number of trees in the ensemble of random forest.
  *f*_*k*_ denotes the function of the k-th tree.
  *X*_*i*_ is the feature vector for observation *i*.
2. EXtreme Gradient Boosting (XGBoost): XGBoost is an optimized implementation of gradient boosting that builds decision trees sequentially, minimizing a specified loss function at each step. It incorporates regularization techniques to prevent overfitting and is particularly effective at capturing non-linear relationships and handling imbalanced datasets (Chen and Guestrin, 2016) (Equation 2).
  (2) $$\begin{array}{l} {ŷ}_{i}=\overset{K}{\underset{k=1}{\sum }}f_{k}\left({\text{x}}_{i}\right),\;f_{k}\in F \end{array}$$
  ŷ_*i*_ represents the predicted value for observation *i*.
  *K* is the total number of trees in the ensemble.
  *f*_*k*_ denotes the function of the k-th tree.
  *X*_*i*_ is the feature vector for observation *i*.
  F is the set of learned decision trees.
3. Support Vector Machine (SVM): SVM aims to find the optimal hyperplane that separates data points of different classes while maximizing the margin between them. By employing kernel functions, SVM can map the data into higher-dimensional spaces to handle non-linear separability effectively (Equation 3).
  (3) $$\begin{array}{l} {ŷ}_{i}=\text{sign}\left(\overset{n}{\underset{j=1}{\sum }}\alpha_{j}y_{j}K\left({\text{x}}_{i},{\text{x}}_{j}\right)+b\right) \end{array}$$
  ŷ_*i*_ represents the predicted value for observation *i*.
  α_*j*_ are the coefficients associated with the support vectors.
  *y*_*j*_ is the class label for observation *j*.
  *K*(**x**_*i*_, **x**_*j*_) is the kernel function evaluated between observations *i* and *j*.
  *b* is the bias term.
  *n* is the total number of support vectors.

### 2.3 Proposed approach for predictive modeling and risk assessment

Following the feature selection phase described in the previous section, the extracted feature sets were used across the selected machine learning models. The dataset was partitioned into three subsets: 20% allocated to the test set and 80% to the training-validation set. Following an adjustment, the training-validation subset was further split, with 80% used for training and 20% for validation, ensuring that model performance was validated on unseen data. To enhance the model results, Bayesian optimization was employed using the Optuna library (Optuna, 2024), which efficiently explores the hyperparameter space to identify optimal combinations for each model (see Table 3). Additionally, an early stopping mechanism with a parameter “early_stopping_rounds=15” was implemented for all models, halting training if no improvement was observed after 15 iterations, thus preventing overfitting. To further evaluate the robustness and generalization capabilities of the models, a k-fold cross-validation approach was implemented. Specifically, a 5-fold cross-validation was employed, dividing the dataset into five subsets of equal size. Each model was trained on four subsets and tested on the remaining one, with the process repeated five times. The results from each fold were averaged to provide a comprehensive assessment of model stability and performance across different data splits. This approach mitigates the risk of overfitting and ensures that the findings are not biased by a particular train-test split. Cross-validation metrics, including precision (Equation 4), recall (Equation 5), F1-score (Equation 6), and the area under the ROC curve (AUC) (Equation 7), were calculated for each fold, further strengthening the reliability of the results.


(4) $$\begin{array}{l} Precision=\frac{TP}{TP+FP} \end{array}$$

(5) $$\begin{array}{l} Recall=\frac{TP}{TP+FN} \end{array}$$

(6) $$\begin{array}{l} F1-score=\frac{2\times Precision\times Recall}{Precision+Recall} \end{array}$$

(7) $$\begin{array}{l} AUC=\int_{0}^{1}TPR\left(fpr\right)dfpr \end{array}$$

To assess the impact of meteorological risk features from Météo-France and those derived from the large language model (LLM), the methodology was executed in two distinct stages. Initially, the analysis included F1 Space: General features combined with risk levels from Météo-France, followed by F2 Space: General features combined with risk levels derived from the LLM. This two-step approach facilitated a comprehensive comparison of the predictive capabilities of the different feature sets in forecasting firefighter intervention activity. By incorporating cross-validation into the evaluation pipeline, this study ensures a more robust and reliable assessment of the models, enhancing the confidence in their generalizability and the conclusions drawn from the comparative analysis.

**Table 3:** Settings hyperparameters.

**Hyperparameter**	**XGBoost**	**Random forest**	**Support vector machine**
max_depth	[2, 17]	[2, 17]	–
min_child_weight	[1, 100]	–	–
n_estimators	[100, 10,000]	[100, 10,000]	–
colsample_bytree	[0.3, 1.0]	–	–
learning_rate	[0.001, 0.3]	–	–
criterion	–	[“gini”, “entropy”]	–
kernel	–	–	[“rbf”, “sigmoid”]
C (regularization)	–	–	[0.01, 10]
gamma (kernel)	–	–	[0.001, 1.0]
class_weight	–	[“balanced”, None]	[“balanced”, None]

## 3 Results

In this study, the collection, processing, and compilation of a comprehensive dataset from various past intervention sources was time-consuming and required substantial effort. Particular attention was focused on meteorological risks, which were either derived from data provided by Météo-France or generated using the LLM, to ensure realistic and reliable results.

Following the training of the selected models and evaluation of their results, the outcomes were presented from various perspectives, ready for in-depth analysis in subsequent sections. Table 4 highlights the predictive performances of each model, focusing on the metrics described in the previous section, for the classification of firefighting activities based on the two feature spaces under study [F1, F2].

**Table 4:** Prediction results for all models based on feature spaces F1 and F2.

**Model**	**Input**	**Precision**	**Recall**	**F1-score**	**AUC**
XGBoost	F1	0.74	**0.76**	0.72	0.85
	F2	0.74	0.75	**0.79**	**0.91**
Random forest	F1	0.73	0.72	0.69	0.82
	F2	**0.75**	0.73	0.76	0.89
Support vector machine	F1	0.70	0.71	0.67	0.80
	F2	0.71	0.71	0.74	0.86

Bold indicates best score by metric.

**Figure 6:**
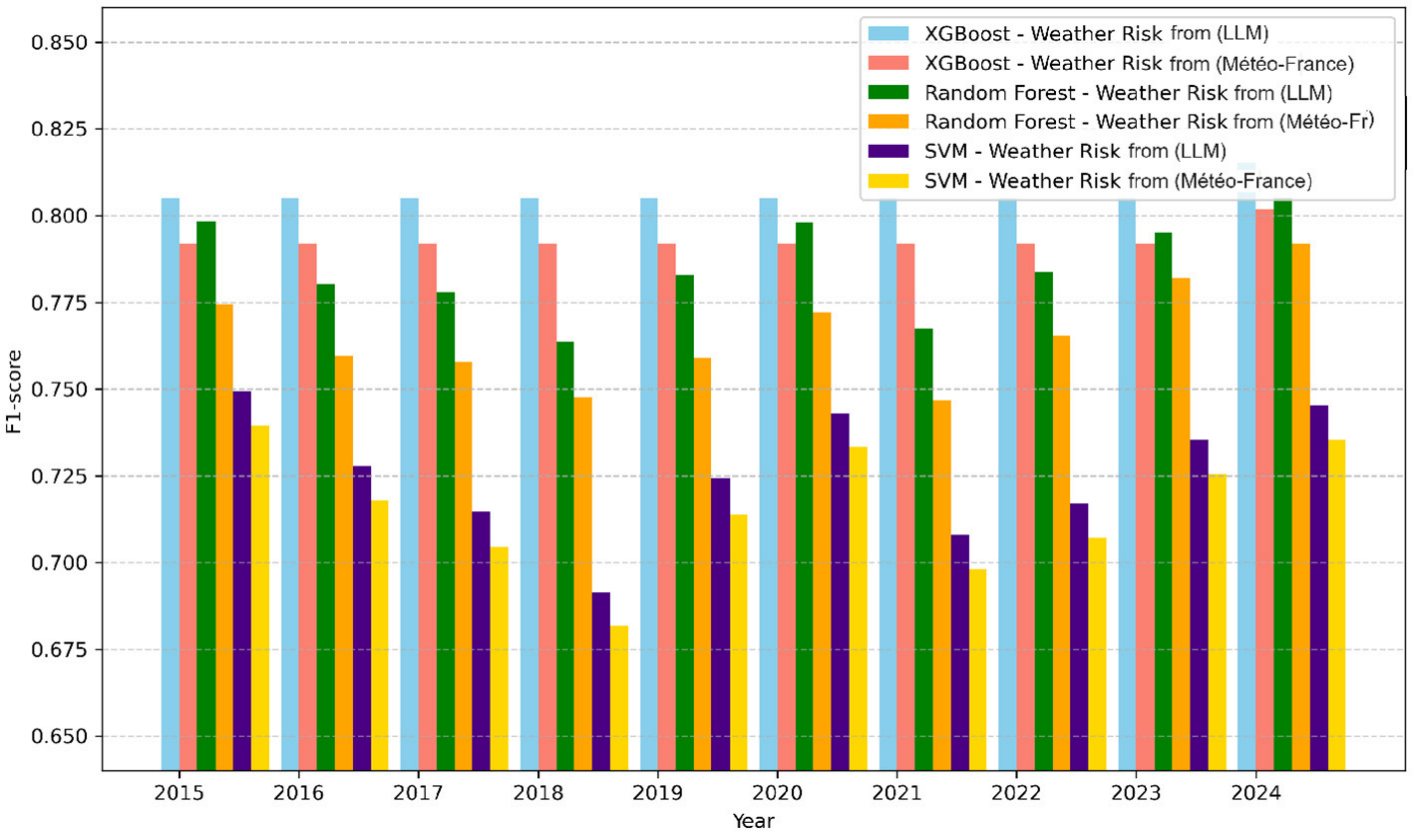
F1-score performance by year for all models (F1 vs. F2).

**Figure 7:**
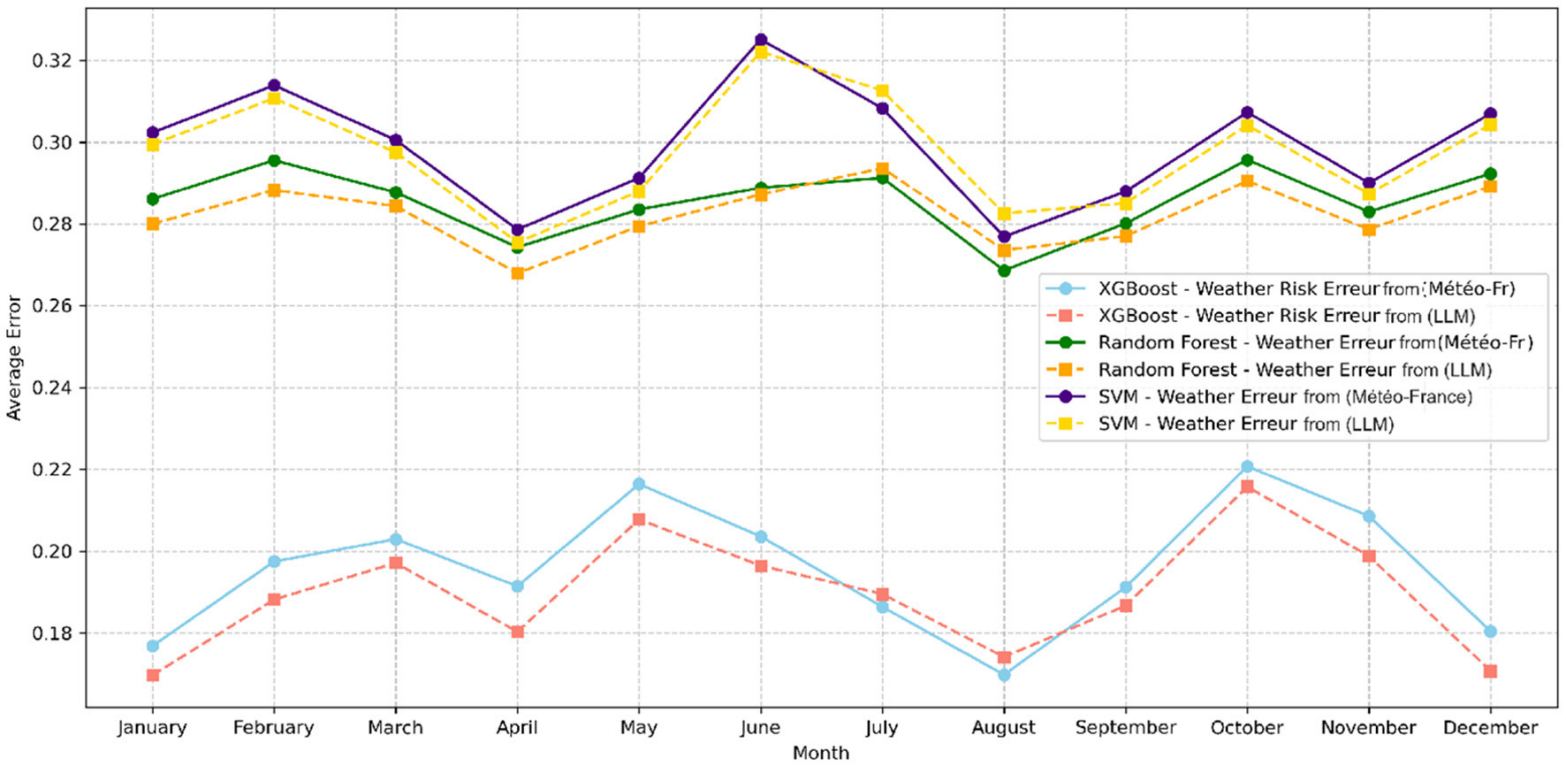
Average prediction errors by month for all models (F1 vs. F2).

**Figure 8:**
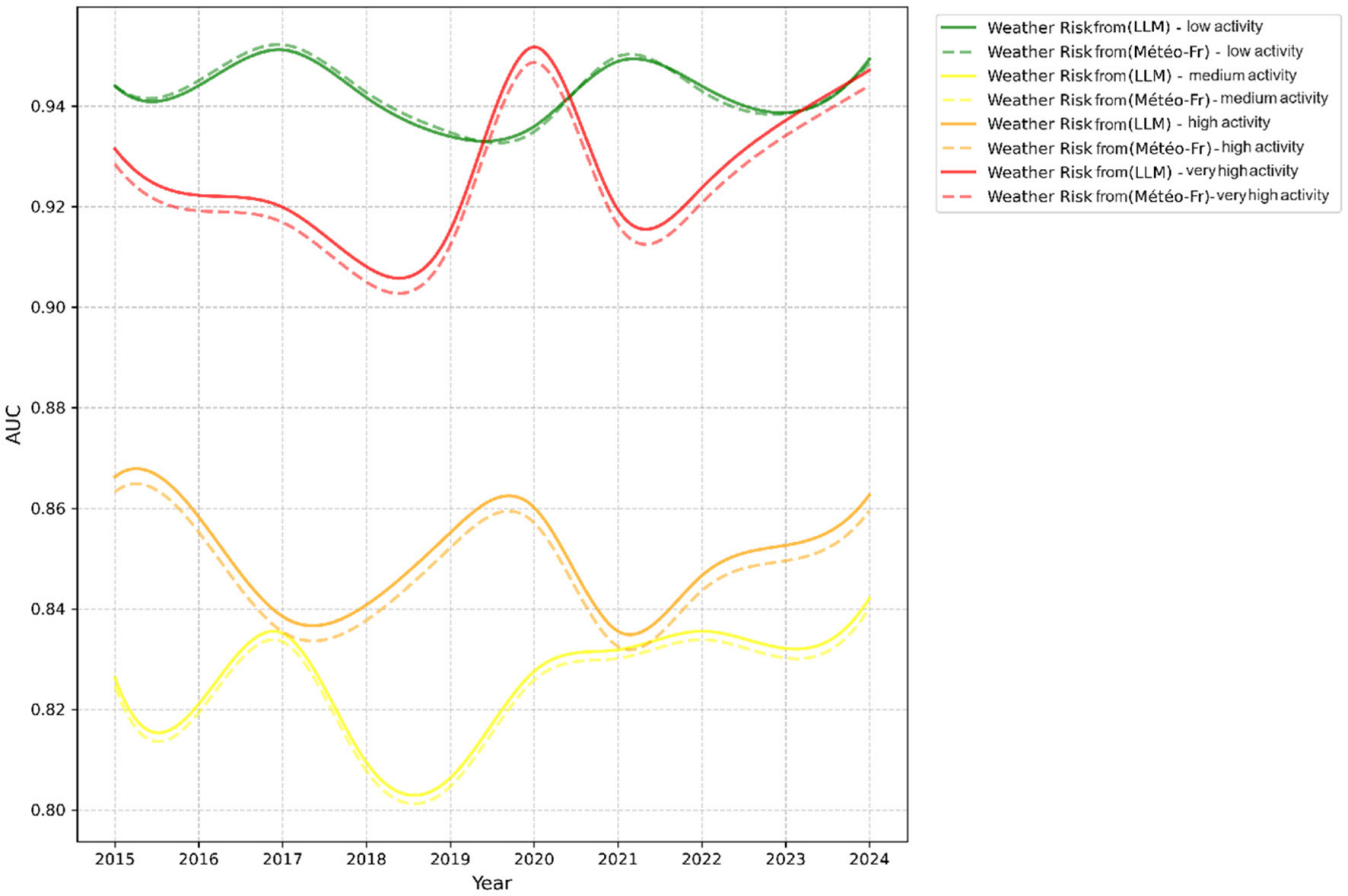
AUC performance by activity over the years.

All models trained with F2 feature space showed reduced prediction errors and overall better performance compared to those trained with F1. The incorporation of cross-validation further reinforced these findings, confirming the stability of the performance improvement associated with the F2 feature space. Figures 6, 7 respectively illustrate the monthly mean prediction errors and the annual F1-score performance for the various models trained with either F1 or F2 feature spaces. Additionally, Figure 8 provides clear evidence on the AUC performance that model XGBoost trained on F2 feature space outperformed those trained on F1 in predicting high and very high firefighter activity levels. However, for medium activity levels, the performance showed no significant differences, and both feature spaces produced similar results for low activity levels.

Finally, Table 5 presents the optimized hyperparameters for each model, obtained through the Optuna framework. These parameters, combined with cross-validation, were crucial in enabling each model to achieve its best predictive performance while ensuring the stability and reliability of the results.

**Table 5:** Best hyperparameters.

**Hyperparameter**	**XGBoost [F1,F2]**	**Random forest [F1,F2]**	**Support vector machine [F1,F2]**
max_depth	[5, 2]	[17, 15]	–
min_child_weight	[10, 9]	–	–
n_estimators	1,000	[500, 490]	–
colsample_bytree	[0.8, 0.7]	–	–
learning_rate	0.1	–	–
criterion	–	“gini”	–
kernel	–	–	“rbf”
C (regularization)	–	–	[1, 2]
gamma (kernel)	–	–	[0.01, 0.03]
class_weight	–	“balanced”	“balanced”

## 4 Discussion

This study aimed to evaluate meteorological risk features derived either from Météo-France or generated using a Large Language Model (LLM) to improve predictions of firefighting interventions over the nine-year period (2015–2024). To achieve this, a detailed methodology was used, involving thorough data preparation, advanced analysis for informed decision-making, and feature selection. Initially, a general set of features was identified using various statistical and machine learning techniques. Subsequently, a second subset of features, focused on meteorological risks either derived from Météo-France or generated by LLM, was extracted, resulting in two feature spaces: F1 and F2. To ensure meaningful and valuable results, three machine learning models [XGBoost, Random Forest, and Support Vector Machine (SVM)] were implemented to evaluate the predictive value of meteorological risk features.

The results demonstrated that models trained on feature space F2, which includes general features combined with meteorological risk features generated by LLM, outperformed others in terms of accuracy and reliability. In particular, XGBoost achieved the highest performance, with an F1-score of 0.79 and an AUC of 0.91 (see Table 4). Furthermore, the performance of this model showed consistent improvement year over year (see Figure 6). This trend suggests that meteorological risks generated by LLM may capture nuanced patterns, likely due to the model's text-based analysis, which is capable of interpreting alarmist or descriptive information. However, this advantage could also reflect greater variability stemming from linguistic subtleties and the absence of direct calibration. A deeper analysis of monthly prediction errors revealed that, although models trained on feature space F2 generally performed better, they underperformed during the summer months compared to those using F1 (see Figure 7). This finding suggests that operational teams should focus on season-specific feature analysis in future studies. This discrepancy could arise due to limitations in the LLM's training data, which may not comprehensively capture seasonal dynamics or low-activity scenarios. Furthermore, the reliance on descriptive prompts may amplify this gap, emphasizing the need for seasonal calibration and prompt refinement. Additionally, F2 inclusion significantly improved predictions for high and very high activity levels. However, for low activity levels, there was little difference between the two feature spaces (see Figure 8). This observation indicates that the detailed textual nature of LLM-generated risk scores might provide reliable insights, particularly for higher-risk scenarios. In contrast, further exploration of weak activity scenarios is warranted.

It is essential to highlight that the LLM's effectiveness depends on the quality of its training data and prompt design. While the study leveraged robust datasets, the validation mechanisms for LLM-generated features remain an area requiring further exploration. Ensuring reliability in critical contexts demands rigorous evaluation protocols, which were not exhaustively discussed in this work. Addressing these aspects would enhance the credibility and operational applicability of such models. From a practical perspective, incorporating cost analysis reveals additional benefits. LLM-based models, despite their computational demands, offer relatively low operational costs when compared to alternative high-resolution meteorological data sources. Moreover, their deployment could lead to more efficient resource allocation, ensuring faster response times and mitigating severe consequences, such as property damage or loss of life, especially in high-risk scenarios. This cost-effectiveness, coupled with improved urgency identification, makes them a valuable tool for firefighting operations. To make the study's findings more tangible, examples of practical impact could include scenarios such as the faster containment of wildfires due to earlier resource deployment or the improved management of simultaneous incidents during peak seasons. These cases underscore the operational significance of integrating LLM-generated features.

Feature analysis is crucial for enhancing operational efficiency in fire intervention planning. A reliable predictive model would enable fire services to better anticipate resource needs, optimize operational logistics, and reduce response times, ultimately improving emergency management, safeguarding lives, and minimizing property damage. However, this study acknowledges certain limitations of applying LLMs in meteorological contexts. These include a reliance on textual data, which can introduce inconsistencies depending on the dataset quality and lack of standardized calibration across different contexts. Furthermore, while LLM-generated features provide valuable insights, their integration into predictive models requires additional validation to ensure robustness. Future research should address these challenges and explore avenues for more comprehensive calibration methods tailored to diverse meteorological datasets. As suggested, incorporating neural networks into the predictive modeling framework, such as multi-layer perceptrons (MLPs) or radial basis function networks (RBFNs), could provide more advanced capabilities. These methods would enable the integration of structured and unstructured data, enhancing the overall accuracy and flexibility of predictions. However, such neural network-based implementations are outside the scope of this study and remain a promising direction for future work. These advanced techniques would complement the existing findings and provide a deeper understanding of the synergy between LLMs and deep learning methods in fire risk assessment and operational planning.

In conclusion, the findings highlight the added value of integrating LLM-generated features into feature analysis, emphasizing the need to tailor approaches to specific scenarios to minimize losses. However, to achieve greater precision and optimization, further research is required. This includes exploring other LLM-based solutions, such as integrating media data for comparative analysis and leveraging deep learning techniques to enhance outcomes.

## 5 Conclusion

This study provides an in-depth analysis to enhance feature selection with a novel approach, optimizing the prediction performance of firefighter intervention activities. It leverages generative AI to extract relevant and optimal meteorological risk information. Using a comprehensive nine-year dataset from SDIS 25 in the Doubs region, France, this research tackles the integration of LLMs into feature generation and evaluates their strengths from multiple perspectives. The findings indicate that models trained on the F2 feature space, which combines general features with meteorological risk scores generated by LLMs, showed superior performance, especially for high and very high intervention activities. However, these models were less effective during the summer season and for low-activity scenarios. This insight could help operational personnel adopt this approach selectively under specific conditions.

As part of ongoing research efforts, a deeper exploration of media-based data is planned to enhance LLM-extracted feature sets. This will involve investigating alternative prediction perspectives and solutions. The primary objective remains to enhance predictive capabilities and optimize resource allocation by extracting the most relevant features for efficient firefighting interventions.

## Data Availability

The raw data supporting the conclusions of this article will be made available by the authors, without undue reservation.
